# Fine-tuning of regulatory T cells is indispensable for the metabolic steatosis-related hepatocellular carcinoma: A review

**DOI:** 10.3389/fcell.2022.949603

**Published:** 2022-07-15

**Authors:** Farooq Riaz, Ping Wei, Fan Pan

**Affiliations:** ^1^ Center for Cancer Immunology, Shenzhen Institutes of Advanced Technology, Chinese Academy of Sciences, Shenzhen, China; ^2^ Chongqing Key Laboratory of Pediatrics, Department of otolaryngology, Children’s Hospital of Chongqing Medical University, National Clinical Research Center for Child Health and Disorders, Ministry of Education Key Laboratory of Child Development and Disorders, Chongqing, China

**Keywords:** regulatory (Treg) cell, forkhead box P3 (FOXP3), hepatocellular carcinoma, metabolic liver diseases, nonalcoholic fatty liver disease -NAFLD, alcoholic liver disease -ALD, NAFLD-associated HCC

## Abstract

The majority of chronic hepatic diseases are caused by nutritional imbalance. These nutritional inequities include excessive intake of alcohol and fat, which causes alcoholic liver disease (ALD) and non-alcoholic fatty liver disease (NAFLD), respectively. The pathogenesis of hepatic diseases is mainly dependent on oxidative stress, autophagy, DNA damage, and gut microbiota and their metabolites. These factors influence the normal physiology of the liver and impact the hepatic microenvironment. The hepatic microenvironment contains several immune cells and inflammatory cytokines which interact with each other and contribute to the progression of chronic hepatic diseases. Among these immune cells, Foxp3^+^ CD4^+^ regulatory T cells (Tregs) are the crucial subset of CD4^+^ T cells that create an immunosuppressive environment. This review emphasizes the function of Tregs in the pathogenesis of ALD and NAFLD and their role in the progression of NAFLD-associated hepatocellular carcinoma (HCC). Briefly, Tregs establish an immunosuppressive landscape in the liver by interacting with the innate immune cells and gut microbiota and their metabolites. Meanwhile, with the advancement of steatosis, these Tregs inhibit the proliferation, activation and functions of other cytotoxic T cells and support the progression of simple steatosis to HCC. Briefly, it can be suggested that targeting Tregs can act as a favourable prognostic indicator by modulating steatosis and insulin resistance during the pathogenesis of hepatic steatosis and NAFLD-associated HCC.

## Introduction

Human beings acquire a sophisticated immune system that actively exists with complex biological mechanisms to defend the host by attacking and destroying the foreign substances (antigen) and transformed or infected cells. Simultaneously, numerous immune regulatory processes are projected to dodge the auto-immune mechanisms resulting against the body’s own tissues. The immune system retains a distinct CD4^+^ cells population, regulatory T cells (Tregs), known to serve essential modulatory roles in immune homeostasis by maintaining peripheral tolerance and controlling the pathological and physiological immune response (Vignali et al., 2008; Lu et al., 2017). These Treg cells assist in limiting the inflammatory responses and abolish autoreactive T cells (Peterson, 2012; Scheinecker et al., 2020).

In 1970, it was first proposed that the presence of thymic-derived suppressor T cells, other than helper T (Th) cells, plays a role in self-tolerance by restricting the effector immune reactions (Gershon and Kondo, 1970). These suppressor T cells were later found responsible for the over-production of immunosuppressive cytokines, such as transforming growth factor β (TGF-β) and interleukin 10 (IL-10), which take part in the immune suppression (O'Garra and Murphy, 1994; Cottrez et al., 2000). Afterward, Sakaguchi et al. (1995), identified the IL-2 receptor α-chain (CD25 molecule) on the surface of these suppressive T cells and named Treg cells. It has been estimated that 5% to 50% of CD4^+^ T cells in the human peripheral blood are comprised of naturally arising CD25^+^ Treg cells that suppress immune activity (Sakaguchi et al., 1995; Gregg et al., 2005). Moreover, CD25^+^CD4^+^ Tregs were determined to express forkhead box P3 (FOXP3), a transcriptional factor which regulated the development and proper functioning of Treg cells (Hori et al., 2003). Therefore, these suppressor T cells can be characterized as CD4^+^CD25^+^FOXP3^+^ Treg cells in murine and humans (Ziegler, 2006).

Multiple mechanisms have been defined for the proper functioning of Treg cells. These include the secretion of cytokines and soluble factors, cell-to-cell contact, and changes in the extracellular milieu to target a diverse population of immune cell populations, such as antigen-presenting cells (APCs), CD8^+^ T killers and cell counterparts of conventional CD4^+^ T cells (Vignali et al., 2008). Emerging evidence suggests the presence of these Treg cells population residing or infiltrating in numerous peripheral organs where they mediate tissue-specific functions. For instance, peroxisome proliferator‐activated receptor‐γ (PPAR-γ) expressing visceral adipose tissue-specific Treg population execute highly distinct functions, including the regulation of numerous genes known to have crucial functions in lipid and glucose metabolism (Cipolletta et al., 2012). Similarly, in muscle and lung, the presence of amphiregulin, a ligand of epidermal growth factor receptor, expressing Treg cells population can directly expedite the tissue repairing process (Burzyn et al., 2013; Arpaia et al., 2015). Foxp3^+^ Tregs play various roles in liver homeostasis and pathogenesis by interacting with other hepatic immune cells and hepatocytes. Hepatocytes have exhibited enclysis ability to engulf CD4^+^ T cells, predominantly Tregs, during hepatic inflammation to regulate T cell population (Davies et al., 2019).

Although the non-lymphoid tissue-specific Tregs molecular signature and functions have been interrogated in numerous investigations, our knowledge of the functions and fundamental biology of these liver-specific Treg cells and how they vary from other non-lymphoid Treg cells and immune cells is yet overlooked. Here, we reviewed the currently available studies about Treg cells that infiltrate the liver, emphasizing the mechanisms in the progression of chronic liver diseases from simple steatosis to hepatocellular carcinoma (HCC).

## Treg cells in hepatic microenvironment

One of the largest internal tissues, the liver, connects with the gastrointestinal tract through the portal vein, which delivers multiple pathogenic and non-pathogenic antigens derived from the gastrointestinal tract (Jenne and Kubes, 2013; Kubes and Jenne, 2018). Therefore, besides enduring non-pathogenic organisms, it plays frontline immunological functions by establishing and escalating specific immune responses (Jenne and Kubes, 2013). As a vast organ, liver retains a predominant population of cells, including T lymphocytes, which are immunologically active and maintain essential immunological functions (Parker and Picut, 2012). The phenotypic characteristics and function of Treg cells differ between intrahepatic and circulatory compartments. This difference is due to the residence of Tregs in the hepatic microenvironment, which are deprived of sufficient oxygen while supplemented with inflammatory cytokines, hormones and metabolic products (Jeffery et al., 2016).

As the gut and liver are connected *via* the portal vein, several environmental factors, including dietary nutrients and metabolites, impact the functionality of Treg cells (Zeng and Chi, 2015). It is evident that gut microbiota and their metabolites strongly affect immune responses (Wu and Wu, 2012). Several microbial metabolites and bile acids have been linked with the expansion and stability of Tregs (Hang et al., 2019; Campbell et al., 2020; Song et al., 2020; Wiechers et al., 2021). Increasing evidence suggests that intake of short-chain fatty acids (SCFAs), metabolites produced by numerous symbionts, improves the proportion of Treg cells in mice treated with antibiotics (Arpaia et al., 2013). Similarly, oral gavage of microbes or SCFAs also activates Treg cells in response to several diseases (Ben Ya’acov et al., 2015; Smith et al., 2013). These gut microbiota and their metabolites also influence the normal physiology and immune response in the liver (Visekruna and Luu, 2021). It can be suggested that microbiota and their metabolites can also play a vital role in stabilizing the Treg cells.

Bile acids, cholesterol metabolites, are immensely produced in the liver through a multiple-step complex process that involves peroxisomal, mitochondrial, and cytosolic enzymes (Russell, 2003). These bile acids are best known to regulate metabolic processes, cellular processes, and the immune system (Chiang and Ferrell, 2019; Fiorucci et al., 2021). Bile acids exert their function by activating numerous receptors, including RAR-related orphan receptor γt (RORγt), liver X receptors α/β (LXRα/β), farnesoid X receptor (FXR), vitamin D receptor (VDR), constitutive androstane receptor (CAR), pregnane X receptor (PXR), and membrane-bound G protein-coupled receptors Takeda G protein-coupled receptor 5 (TGR5) (Keitel et al., 2019; Shin and Wang, 2019; Cai et al., 2021). Among these receptors, FXR and TGR5 have been acknowledged as legitimate targets for treating metabolic-associated NAFLD (Arab et al., 2017). Activation of these receptors also influences and shapes the innate immune response, thus playing critical roles in the progression and development of NAFLD-related HCC (Schubert et al., 2017). Increasing evidence indicates that bile acids and their receptors also influence the adaptive immune system (Campbell et al., 2020; Song et al., 2020); however, the role of these receptors in adaptive immune response, especially in liver resident Tregs, is not studied well. A recent study identified isoallo-LCA and 3-oxo-LCA as metabolites of lithocholic acid (LCA). Among these, 3-oxo-LCA directly binds to the RORγt and suppresses the differentiation of Th17 cells. Meanwhile, isoallo-LCA stimulates mitochondrial ROS production and promotes Treg differentiation by increasing FOXP3 expression (Hang et al., 2019). Interestingly, obeticholic acid (OCA), FXR agonist, was approved by FDA for the treatment of primary sclerosing (Ali and Lindor, 2016). On the other hand, OCA ameliorates fibrosis and NASH; thus, it exerts beneficial effects by reducing hepatic cirrhosis (Shah and Kowdley, 2020). However, the effect of OCA and other bile acid molecules on the population and function of FOXP3^+^CD4^+^ cells is not studied.

It has been documented that fat-soluble vitamins are highly prevalent in the liver (Stacchiotti et al., 2021). A metabolite of vitamin A, all-trans retinoic acid (RA), plays a role in the growth, differentiation and proper functioning of immune cells, including Treg cells (Liu et al., 2015). In the liver, RA is produced by stellate cells, which increases the Foxp3 in CD4^+^ T cells and takes part in the development (Dunham et al., 2013), functioning and stability of the Treg cells during the inflammatory microenvironment (Zhou et al., 2010; Lu et al., 2014). TGF-β is considered one of the essential immunosuppressive cytokines that activate the population of CD4^+^ CD25^+^ FoxP3^+^ Treg cells (Kanamori et al., 2016). RA can directly influence and promote the TGF-β-dependent differentiation of naive T cells into the FoxP3^+^ Treg cells (Mucida et al., 2009; Martínez-Blanco et al., 2021).

Besides the TGF-β in an inflammatory hepatic microenvironment, several other pro-inflammatory cytokines, including IL-1, IL-12, IL-6, IL-8, and TNFα, are also present. However, it was reported that IL-2 is deficient, which is necessary for the overall survival of Treg cells (Chen et al., 2016a). Activation of APCs is known to secrete these pro-inflammatory cytokines (Blanco et al., 2008). These intrahepatic resident Treg cells also rely on the APCs for their differentiation, proper functioning, and survival. Tregs interact with APCs by binding with CD80/86 through CD28/CTLA-4, engaging with MHC Class II through TCR in the presence of antigen, and responding to APC secretory cytokines through cytokine receptors (Goddard et al., 2004; Lai et al., 2007). However, TGF-β, in combination with higher IL-6, generates IL-17 producing Th17 cells from naïve T cells, which further suppresses the induction of Treg cells (Bettelli et al., 2006). In addition, hepatocytes also play an important role in the differentiation of Foxp3^+^ Tregs upon TCR stimulation from the CD4^+^ T cells *via* Notch-signaling (Burghardt et al., 2014). Resident Treg cells, even present in slightly lower regulatory potential, acquire an intact functional ability and affirm short-term lineage during culturing conditions that imitate and maintain the intrahepatic microenvironment (Chen et al., 2016a).

Immune metabolic pathways have been well studied for the activation, differentiation, survival, and proliferation of the immune cells, including metabolically active Treg cells (O'Neill et al., 2016; Wang et al., 2019). The expression of Foxp3, a pivotal transcription factor of Treg, is known to involve in metabolic pathways, such as glycolysis and fatty-acid oxidation (Fontenot et al., 2003; Gerriets et al., 2016; Angelin et al., 2017). During inflammatory circumstances, the ratios of metabolic supply-and-demand dramatically alter. Inflammatory lesions induce the level of hypoxia-inducible factor-1α (HIF-1α) in the tissues deficient in enough oxygen. HIF-1α plays a vital role in enhancing the population and suppressive roles of thymic Treg (Ben-Shoshan et al., 2008; Clambey et al., 2012) by directly binding the promoter region of FOXP3 (Clambey et al., 2012). However, during an oxygen-deficient environment, the ablation of mTOR signaling is found to be involved in the elimination of the HIF-1α functions (Land and Tee, 2007). Activating transcription factor 3 (ATF3), a member of ATF/CREB transcriptional family, is induced at early inflammatory responses by cytokines, i.e., IFN-α, IFN-β, IFN-γ and IL-4 (Farber, 1992; Drysdale et al., 1996). The absence of ATF3 escalated the mTOR-dependent induction of the HIF-1α, which minimized the Foxp3^+^ Treg cells (Zhu et al., 2018). Therefore, the hypoxic anti-inflammatory process induces HIF-1α that improves the population and functions of Treg cells by strengthening their effectiveness and reducing the proliferation of effector cells (Ben-Shoshan et al., 2008).

**FIGURE 1:**
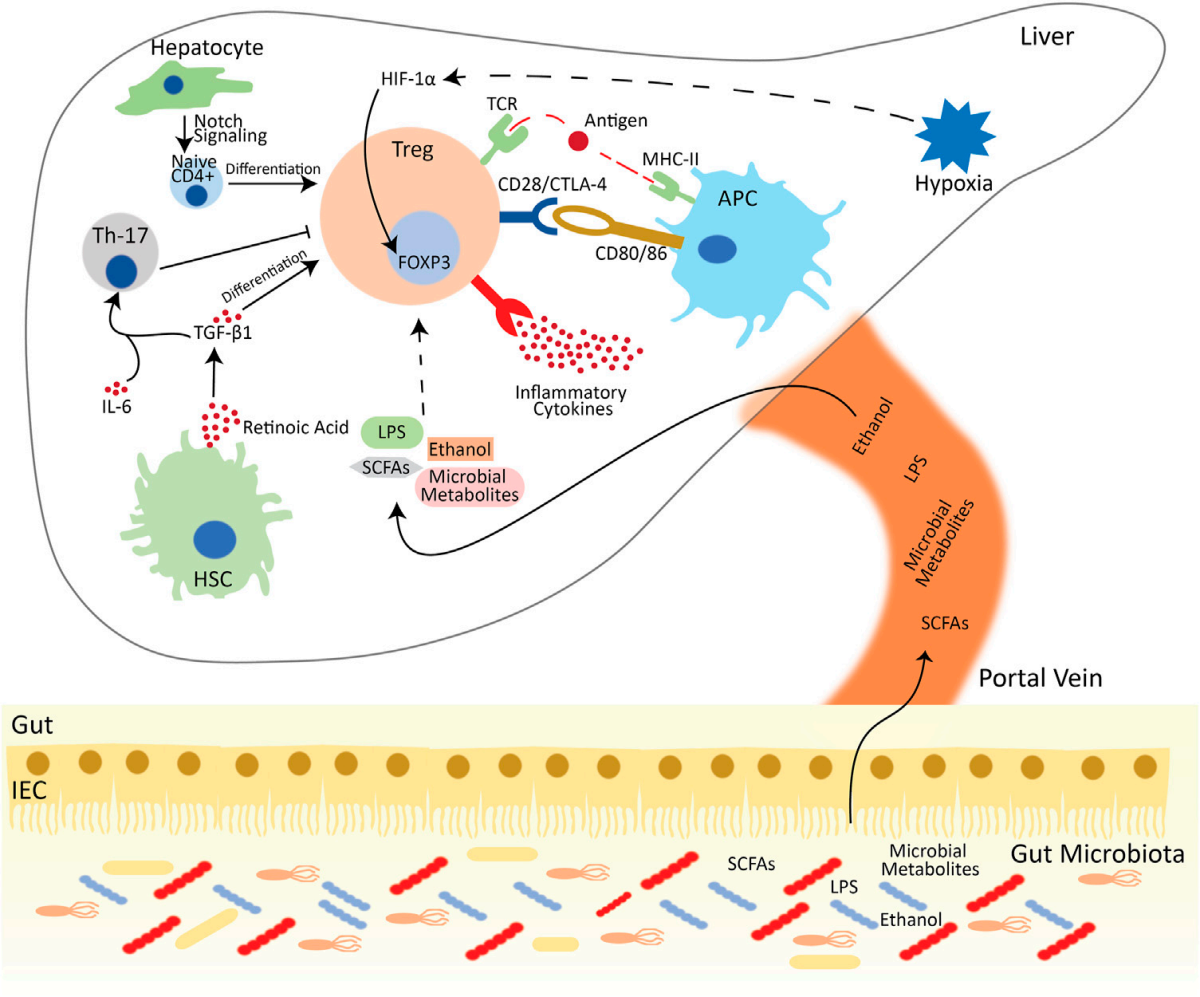
Treg mediated immune suppression in the hepatic microenvironment. Tregs are activated by interacting with the MHC-II on the surface of APCs in the presence of antigen. Tregs also suppress conventional T cells by interacting with pro-inflammatory cytokines and pairing with CD80/86 *via* CTL-4/CD28 and deprive the co-stimulatory signal to responder T cells. Besides the APC, Tregs also interact with other hepatic cells. Hepatocytes differentiate the naïve CD4^+^ cells into FOXP3^+^ Tregs *via* notch signaling. HSCs release retinoic acid, which activates the TGF-β signaling and aids Tregs differentiation. Meanwhile, in the presence of IL-6, TGF-β activates Th-17 cells, which reduces the activation and development of Tregs. Additionally, in oxygen-deprived environment, HIF-1α directly interacts with the FOXP3 and enhance the population of Tregs. Besides the intrahepatic regulation of Treg development and functions, gut microbiota and their metabolites also influence the Treg functions. Abbreviations: APC, Antigen presenting cell; Treg, regulatory T cell; Th-17, T helper 17 cell; HSC, hepatic stellate cell; IL, interleukin; CTLA4, cytotoxic T lymphocyte-associated antigen 4; DC, dendritic cell; CD, cluster of differentiation; TGF-β1, transforming growth factor-beta 1; MHC, major histocompatibility complex; IEC, intestinal epithelial cells; LPS, lipopolysaccharide; SCFA, Short-chain fatty acids.

Conclusively, it is evident that hepatic immune responses and Treg population and functionality are greatly dependent on the hepatic microenvironment (Figure 1). This hepatic microenvironment has an excessive hypoxic atmosphere due to higher blood supply, especially around zone 3 (Jeffery et al., 2016). Meanwhile, other environmental signallings, including environmental factors, microbial metabolites and metabolic pathways, also influence the transcriptional programming of Foxp3 and the functional plasticity of Treg cells.

## Functions of Tregs in metabolic-associated chronic hepatic diseases

**FIGURE 2:**
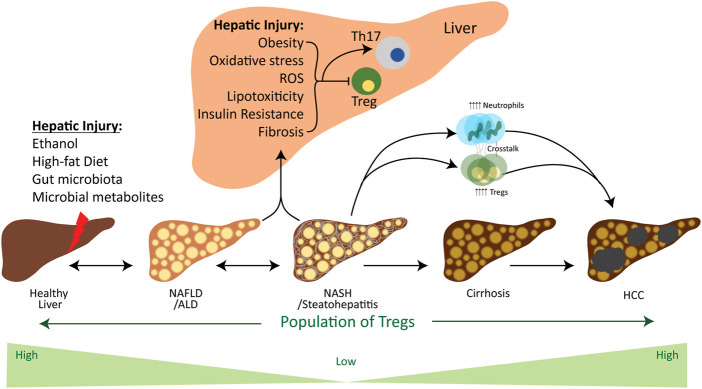
Influence of factors involved in the chronic liver disease spectrum on the Treg cells. Hepatic injury is caused by various environmental stressors, including ethanol, a high-fat diet and gut microbiota and their metabolites. These stressors initiate the simple steatosis and lead to steatohepatitis and cirrhosis in the liver, which finally progresses towards the tumor development. In the beginning, environmental stressors induce hepatic oxidative stress, ROS, lipotoxicity, insulin resistance, fibrosis and obesity, which are the pathogenic factors for ALD, NAFLD, NASH and HCC. These factors considerably contribute in downregulating the activation of Tregs and their population for the development of steatosis and fibrosis. However, the progression of NASH towards the tumor development increases the population of Tregs to establish a pro-tumor microenvironment. Meanwhile, adaptive and innate immune systems communicate with each other through Tregs and neutrophils, which aid in the progression of NAFLD-associated HCC. Abbreviations: Treg, regulatory T cell; Th-17, T helper 17 cell; NAFLD, Non-alcoholic fatty liver disease; ALD, alcoholic liver disease; NASH, non-alcoholic steatohepatitis; HCC, hepatocellular carcinoma.

As mentioned earlier, the human liver acquires a large amount of blood supply, ∼70%–80%, from the portal vein, which is intensified with numerous metabolites and nutrients (Balmer et al., 2014). Therefore, the liver-resident immune cells, e.g., APCs, Tregs and T effector cells (Teffs), are continuously exposed to numerous signals which significantly impact their activation and alter their functions. Besides the effects of metabolites and nutrients on Tregs, innate immune cells and pro-inflammatory cytokines also impact the Tregs (Figure 2). Reported data on the immunosuppressive functions of Tregs suggest that intrahepatic suppression/overexpression or inadequate regulation of Tregs contribute to the onset of various diseases, including chronic hepatitis B&C virus (Cabrera et al., 2004), HCC (Unitt et al., 2005), autoimmune hepatitis (Longhi et al., 2004), alcoholic liver disease (ALD) (Matos et al., 2013), non-alcoholic liver disease (NAFLD) (Rau et al., 2016), primary biliary cirrhosis (Lan et al., 2006), acute rejection after liver transplant (Demirkiran et al., 2006). Here, we will be focusing on the function of Tregs in metabolic-associated chronic hepatic diseases, including ALD, NAFLD, NASH, and NAFLD-associated HCC.

## Role of Treg in alcoholic liver disease

Alcohol abuse, an emerging and alarming health concern with high mortality worldwide, encompasses a broad range of injuries. These include alcoholic liver disease (ALD) with mild steatosis to steatohepatitis, fibrosis, and cirrhosis, leading to hepatocellular cancer (O'Shea et al., 2010; Breitkopf et al., 2009). It has been estimated that excessive alcohol intake is responsible for up to 4% of the yearly deaths (Singal and Anand, 2013). Ample evidence suggests that innate and adaptive immune responses are entailed in the development, pathogenesis and progression of ALD (Mille r et al., 2011; Albano, 2012; Gao et al., 2019). Alcoholic hepatic injury recruits peripheral immune-related cells, including infiltrating monocytes, neutrophils and T lymphocytes (Chedid et al., 1993; Nagy, 2015). Excessive exposure to alcohol or chronic ALD leads to the dysregulation of the balance between these immune cells and disruption of the immune activity. It contributes to the development of the unresolved inflammation features of ALD.

The role of inflammatory response activation and aberrant immune responses in worsening the ALD and its outcome has been extensively studied. It is generally believed that mild or excessive ethanol abuse may modify the immune responses and functions (Szabo et al., 2011; Azizov et al., 2020; Luck et al., 2021). Ethanol impairs the functions of APCs and monocytes, decreases the proliferation of T cells and interferes with the expression of adhesion molecules (Szabo and Mandrekar, 2009). During the progression of ALD, there is an increase in the production of pro-inflammatory chemokines/cytokines by the liver resident macrophages, i.e., kupffer cells (KC) (Slevin et al., 2020). Meanwhile, damaged hepatocytes may produce several antigens to initiate the intrahepatic immune responses, resulting in massive intrahepatic inflammatory cell recruitment, including Treg cells (Viitala et al., 2000).

With the advancement in experimental techniques, multiple T cell subtypes have been identified. It has been recognized that alcohol exposure impacts the T cells phenotypes and Treg cells population (Matos et al., 2013). Earlier studies elucidating the molecular mechanism underlying the progression and development of ALD illustrated the presence of CD4^+^ and CD8^+^ cells in the liver biopsies from ALD patients (Chedid et al., 1993). The population of peripheral blood CD4^+^/CD25^+^ Treg cells is not altered (non-significant increase) in chronic alcoholic patients. Still, it significantly decreases with the increased inflammatory cytokines in patients with alcoholic hepatitis (AH) (Almeida et al., 2013). A similar relative and absolute Treg population in AH, regardless of chronic hepatopathy symptoms, concluded that decreased CD4^+^/CD25^+^ Treg cells in AH depend on the acute hepatic inflammation (Almeida et al., 2013).

Similarly, Treg cells are most likely to participate in the pathogenesis of viral hepatitis in individuals with alcohol abuse. It was evident by the enhanced CD4^+^ FOXP3^+^ and CD25^+^ FOXP3^+^ Treg cell subtypes which were prompted in mice immunized with DCs, isolated from ethanol-fed mice, and loaded with HCV core (Ortiz and Wands, 2014). In contrast to decreased circulatory CD4^+^/CD25^+^ Treg cells in alcoholic patients (Almeida et al., 2013), another study reported an increased population of circulatory CD4^+^/CD25^+^ Treg cells in alcoholic patients (Ribeiro et al., 2017), suggested the role of Treg cells in reducing the detrimental effects of excessive alcohol intake on the liver. Meanwhile, it has been investigated that inflammatory immune response in ALD has resulted from the increased Th17 population (Kasztelan-Szczerbińska et al., 2015), and Treg cells reduce the development and differentiation of Th17 cells by modulating the levels of anti-inflammatory IL-10 and TGF-β cytokines (Chaudhry et al., 2011; O'Garra and Vieira, 2004; Lee, 2018). Likewise, the increased distribution of Treg cells close to the portal tract in the inflamed human liver demonstrates the close association with the suppression of immune response (Oo et al., 2010). Therefore, it can be suggested that an increased Treg cells population in patients with extreme ethanol might reduce the alcoholic hepatic inflammatory responses.

As previously described, the liver is directly connected to the gut and is continuously susceptible to harmless antigens and byproducts of gut bacteria. Currently available studies have discovered that alterations in the intestinal microbiome serve an important regulatory role in initiating ALD in humans and murine (Yan et al., 2011; Mutlu et al., 2012). Likewise, excessive alcohol intake may damage microbiome balance, distort the intestinal barrier, and lead to a dysfunctional liver and other vital organs (Bishehsari et al., 2017). Alcohol-induced intestinal barrier disruption helps the endotoxins, including lipopolysaccharide (LPS), and cytokines, including IL-6 and TNFα, to translocate into the liver and interact with hepatocytes and immune cells (Bala et al., 2014; Bishehsari et al., 2017; Zheng and Wang, 2021), which play pivotal roles in ALD and hepatic inflammation. Evidence of the ethanol-dependent modifications in the expression of CD4^+^ T Cell subsets in LPS-stimulated peripheral blood mononuclear cells suggests that ethanol impedes the Foxp3 kinetics and the production of IL-1 and TNF-α after the LPS challenge, thereby affecting the Treg/Th17 cells balance (von Haefen et al., 2011).

Ethanol intake damages the liver by reducing the population of Treg cells while increasing the Th17 cells population along with the production of IL-17 and increasing the intestinal permeability by reducing the expression of tight junction proteins (Wang et al., 2011; Chen et al., 2016b). Probiotic supplementation is well known to improve the functions of the intestinal barrier by improving the expression of tight junction proteins and exerting protective effects in response to damaging factors, including alcohol abuse (Rao and Samak, 2013). Lactobacillus rhamnosus GG (LGG) is a probiotic that strives to treat and prevent various diseases by stimulating immune responses (Segers and Lebeer, 2014). A recent study showed that LGG supernatant supplementation ameliorates ALD by improving the population of Treg cells and decreasing the Th17 population (Chen et al., 2016b).

Alcohol consumption, either acute or chronic, lowers the antigen presentation by DCs, and reduces the T-cell proliferation and activation by affecting the levels of costimulatory molecules (Eken et al., 2011). Hepatic resident APCs activate the Treg cells from naive CD4^+^ precursors (Bamboat et al., 2009). In ALD-cirrhosis patients, diminished levels of circulatory IL-1β, IL-6, IL-12, and TNF-α inflammatory cytokines, along with fewer number of circulatory DCs have been observed (Laso et al., 2007). Excessive alcohol consumption impairs the production of cytokines IL-6, IL-12, IL-17A and IFN-γ from APCs which are basically involved in initiating the adaptive immune response (Heinz and Waltenbaugh, 2007). Generally, ethanol diminishes the expression of stimulatory molecules on the surface of DCs, impairs their ability to prime T cells, affects the activation of naive T cells, and restricts the development of allogeneic T cells. Upon CpG stimulation, hepatic DCs collected from ethanol-fed mice exhibited reduced functional maturation (Lau et al., 2006). It implies that the alcohol-induced dysfunctional DCs might serve as a protective mechanism for immunosuppression by excessive alcohol drinking (Szabo, 1999; Lau et al., 2006) and may impair the Treg population.

Meanwhile, cell-cell interaction between DCs and Treg cells depends on the MHC-II for activation of Treg cells. Individuals with chronic alcohol intake may show lower MHC II-dependent T cell response (Chang and Norman, 1999). It can be advocated that besides the exclusive effects of alcohol on the cells responsible for innate immune response, early consequences on the adaptive immune system by alcohol contribute to the AH and ALD. Similarly, dysfunctional Treg cells are well known to contribute to the development and progression of these alcoholic diseases. However, the influence of alcohol on the roles of liver resident Treg cells is not studied well. Therefore, further investigations are necessary to improve our understanding of mechanisms underlying ALD and provide aid in finding novel therapeutic targets to treat AH and ALD.

## Role of Treg in non-alcoholic fatty liver disease

Non-alcoholic fatty liver disease (NAFLD), affecting one-third of the population, is known for the presence of hepatic steatosis, which accelerates a series of hepatic diseases ranging from non-alcoholic fatty liver (NAFL) to non-alcoholic steatohepatitis (NASH) and progress to cirrhosis and hepatocellular carcinoma (HCC) (Younossi et al., 2016; Younossi et al., 2018). Hypothetically, the progression of NAFLD has been illustrated in a “multi-hit” manner, which initiates the accumulation of lipids in hepatocytes. Afterward, an increase in free fatty acids secretion from adipocytes, oxidative stress, decreased adiponectin, and increased pro-inflammatory cytokines (resistin, leptin, TNFα, and IL-6) collectively prompt the development of hepatic steatosis and inflammation (Starley et al., 2010). Meanwhile, several other factors, including TGF-β1-dependent collagen deposition, macrophage activation, hepatic reactive oxygen species (ROS), metabolically active natural killer T cells (NK-T) and CD8^+^ T cells, and imbalance between Th17 and Treg, take part in the disease progression beyond NAFL (Wolf et al., 2014; Paquissi, 2016).

Increasing evidence suggested the close association of NASH with activated innate immune response in mice (Tosello-Trampont et al., 2012) and humans (Malehmir et al., 2019). However, the role of adaptive immunity and Treg cells in NASH hasn’t been studied well. A recent clinical study showed the low population of resting Tregs in the peripheral blood of NASH patients and an increase in intrahepatic Th17 cells (Rau et al., 2016), suggesting that higher Th17/rTreg is engaged in NAFL to NASH progression. Another study indicated that NAFLD-related severe hepatic inflammation in children was linked with higher intralobular Foxp3^+^ lymphocytes, while adults exhibited decreased Foxp3^+^ and higher IL-17A^+^ lymphocytes in portal/periportal (P/P) tracts (Cairoli et al., 2021). Similarly, the high-fat diet (HFD)-induced NAFLD model showed an elevated population of intrahepatic Th17 cells. This increased Th17 and IL-17 are linked with the development of steatosis and the expression of pro-inflammatory cytokines (Tang et al., 2011). Subsequently, increased Th17 in liver during the chronic liver injuries exhibits a decreased proportion of Treg cells with the increased IL-6, IL-17 and IL-23 (He et al., 2017; Khanam et al., 2019).

Insulin resistance (IR), lipotoxicity and adipose inflammation are the hallmarks of NAFLD. Generally, lipotoxicity accelerates the pathogenesis of NAFLD by aggravating hepatic inflammation, adipose tissue inflammation and IR (Manco, 2017). It has been implicated that CD4^+^ T cells, especially Tregs, play critical roles in the regulation of IR, adipose inflammation and obesity. For instance, Tregs repress the immune response, whereas Th1 and Th17 cells enhance adipose inflammation (Bluestone et al., 2009; Newton et al., 2016). Upon activation, resting naive CD4^+^ T lymphocytes are differentiated into Tregs and Teffs to and employ immunological responses. Obesity and IR affect the Tregs cells by suppressing their differentiation or impairing their functions (Feuerer et al., 2009; Cipolletta et al., 2012; Wagner et al., 2013). It was found that maintenance of Tregs in VATs of HFD-induced obese animal model significantly reduces adipocyte size and decreases the body weight gain and visceral adipose tissue weight; thus, impairing Tregs worsens HFD-induced obesity and IR in mice (Tian et al., 2011; Cipolletta et al., 2012).

Several cellular metabolic activities, including inflammation, stress responses, and cell survival, are responsible for the oxidative stress and production of ROS within the cells (Pizzino et al., 2017). ROS production promotes hepatic inflammation, fibrogenesis and lipotoxicity in NAFLD (Delli Bovi et al., 2021). Lack of fatty acid β-oxidation along with intensive lipogenesis cause the excessive accumulation of triglycerides within the hepatocytes. During the process of NASH, increased ROS combines triglycerides and leads to IR and hepatic steatosis (Mansouri et al., 2018). Oxidative stress in the liver exerts detrimental effects on the hepatic Tregs. It was observed that oxidative stress induces Treg apoptosis and leads to their deletion within the steatotic liver (Ma et al., 2007), and consequently increases hepatic inflammation and avitivates TNF-α signaling pathway. This results in further hepatic injury, including the progression of simple steatosis to steatohepatitis, especially when liver is exposed to endotoxins, e.g., LPS, which can be delivered to liver or endogenously produced by gut microbiota (Ma et al., 2007; An et al., 2021). Moreover, adoptive transfer of Tregs have decreased HFD-induced intrahepatic TNF-α signaling and diminished the LPS-induced hepatotoxicity (Ma et al., 2007).

As mentioned earlier, activation of Tregs and their ability to perform suppressive functions are somehow dependent on the MHC-II. Liver sinusoidal endothelial cells (LSECs) and KCs are the key MHC-II expressing hepatic APC populations, which display antigen to Tregs and other CD4^+^ T cells (Wiegard et al., 2005). These Treg cells stimulated by KC and LSEC suppress the other CD4^+^ T cells proliferation (Wiegard et al., 2005). Similarly, hepatocytes can express MHC-II during hepatic inflammation, which helps them contribute to inflammatory immune responses by overcoming Treg suppressive functions during microbial antigenic signals (Herkel et al., 2003; Wiegard et al., 2005). The inflammatory hepatic microenvironment, i.e., TNF-α, IFN-γ and oxidative stress induced by KCs and DCs impair the survival and induce the apoptosis of Foxp3^+^ Treg cells during the apoptosis of hepatocytes and NASH (Ma et al., 2007; Roh et al., 2018). However, another study reported that the population of hepatic Tregs enhanced during the NASH, and depletion of Tregs can significantly inhibit the development of HCC from choline-deficient, high-fat diet feeding and diethylnitrosamine injection-induced NASH model (Wang et al., 2021). These opposing findings describing the functions of Tregs in NASH could have resulted from the different NASH models, or there is a probability that Tregs exert contrasting functions during the early and late NASH.

KLF10 is a well-known responsive transcription factor of TGF-β1, which regulates the functions and differentiation of Teffs and Tregs (Cao et al., 2009). A recent study concluded that the expression of KLF10 is significantly reduced in Teffs and Tregs isolated from peripheral blood and spleen of HFD-induced and obese mice (Wara et al., 2020). It is known that diet-induced obesity and liver diseases increase the immune cell accumulation in the liver and aggravate hepatic inflammation and lipid metabolic dysfunction (Dallio et al., 2021). CD4^+^ T cell-specific KLF10 deficiency leads to inflammation in adipose tissue, IR, obesity and the onset of NAFLD with impaired Treg accumulation (Wara et al., 2020). However, adoptive transfer of Tregs in CD4^+^ T cell-specific KLF10 deficient mice impede obesity, IR, adipose tissue inflammation and fatty liver phenotype (Wara et al., 2020). Overall, Foxp3^+^ Tregs exert protective roles during the progression of NAFLD from simple steatosis to steatohepatitis. Therefore, it can be concluded that despite the activation and marginal clonal expansion of hepatic T cells in NASH, the increased population of Treg counterbalance these effects. Meanwhile, the adoptive transfer of Treg in NASH aggravates the disease severity (Dywicki et al., 2022). Overall, the current understandings of the protective function of Tregs are still limited and need additional investigations to provide aid in treating NAFLD and NASH through Treg targeted therapy.

## Role of Treg in NAFLD-associated HCC

HCC is deemed a major histological type of primary liver cancer which accounts for approximately 75% of all hepatic cancers (McGlynn et al., 2015). Leading evidence suggests that a high incidence of NAFLD and the ultimate progression of liver diseases are the leading causes of HCC. NASH leads to the hepatocytes’ death and compensatory proliferation, and converts the mild fibrosis to advanced fibrosis with elevated levels of TNFα, TGF-β1 and IL-18, which increase the risks of HCC (Anstee et al., 2019). The tumor microenvironment comprises cancer cells, immune cells and their mediators, and pro-inflammatory cytokines and chemokines (Gallimore and Simon, 2008). In the liver, the pathogenesis of NASH-associated HCC exclusively depends on the intrahepatic inflammatory and immune responses, autophagy, oxidative stress and DNA damage (Wolf et al., 2014; Anstee et al., 2019). Immune evasion of cancer cells is regulated by various immune suppressor mechanisms, which involve different subsets of immune cells and contribute to the initiation and progression of HCC (Greten et al., 2015). Meanwhile, lymphocytic infiltrate at the tumor site decreases the risk of a recurrent tumor and increases the overall survival rate (Greten et al., 2013). However, the role of T cells, especially Tregs, in NASH-associated HCC is not understood well. Striking evidence advocated that the Tregs population increases in peripheral blood and tumor tissues collected from HCC individuals (Guo et al., 2014). Therefore, researchers believe that increase in Tregs may exert adverse effects on the HCC disease prognosis (Wilke et al., 2010), and an augmented ratio of effector CD4^+^/Treg cells represent a better prognosis for HCC (Kalathil et al., 2019).

Several studies have determined the critical role of CD4^+^ T cells in the initiation and progression of HCC. CD4^+^ T cells generally impede the HCC initiation and progression, thereby contributing to the tumor regression (Rakhra et al., 2010). Tregs adversely affect the local immune microenvironment (Nishida and Kudo, 2017). FOXP3^+^ Tregs exert immunosuppressive functions in the tumor settings by restricting the development and activation of anti-tumor effector cells and facilitating the tumor immune escape (Khazaie and von Boehmer, 2006). Thus, elevated population of CD4^+^ CD25^+^ FoxP3^+^ Tregs promotes the disease initiation and progression by impairing the functions of effector CD8^+^ cells (Fu et al., 2007; Gao et al., 2007; Shi et al., 2018). Earlier studies have indicated that Tregs could regulate the differentiation and development of T cells by secreting anti-inflammatory IL-10 and TGF-β1 or repressing the IFN-γ and the T cells proliferation, thereby inhibiting their immune function (Bergmann et al., 2011). Meanwhile, HCC tumors themselves secret TGF-β1, which serves as the foremost vital source of TGF-β1 in HCC patients (Wang et al., 2016). This TGF-β1 could be a major factor in activating the regulatory phenotype of Tregs and maintaining their biological functions (Marie et al., 2005).

The functional heterogenicity of CD4^+^ T cells embraces Teff and Treg cell functions depending on their differentiation (Sallusto and Lanzavecchia, 2009). HCC pathogenesis greatly depends on the selective loss of hepatic resident CD4^+^ T cells, which accelerates the progression of HCC from NAFLD liver (Ma et al., 2016). It is obvious that IFN-γ secreting cytotoxic CD4^+^ Th1 cells monitor and clear the premalignant senescent hepatocytes (Kang et al., 2011). However, FOXP3^+^ Tregs inhibit the proliferation and function of Th1 and other Teffs. Therefore, despite the decreased population of total CD4^+^ in NASH liver, an enhanced population of Tregs has been observed, which aggravates the inflammation in NASH by establishing the pro-tumorigenic settings and leading to the initiation of the NASH-HCC malignant process (Wang et al., 2021).

Yes-associated protein-1 (YAP1) is a transcriptional coactivator and downstream effector of the Hippo signaling pathway (Manmadhan and Ehmer, 2019). Several studies have reported the positive correlation of YAP1 with the severity of hepatocyte injury and the progression of NAFLD and NASH (Chen et al., 2018; Salloum et al., 2021). Moreover, inhibiting the YAP1, reported as an independent prognostic marker and associated with the disease-free survival HCC patients (Xu et al., 2009), restores hepatocyte differentiation, and reduces the tumor development and the advancement of HCC (Fitamant et al., 2015). Besides the direct evidence of YAP in the development of NAFLD-HCC, it was investigated that YAP is necessary for the differentiation and immunosuppressive functions of Treg cells (Fan et al., 2017; Ni et al., 2018).

Mounting evidence indicated the potential role of gut microbiota in modulating T-cell immunity directly or *via* their metabolites, including SCFAs (Asarat et al., 2016). Microbial dysbiosis leads to the generation of excessive amounts of SCFA, especially butyrate, which aid in setting the tumor microenvironment (Singh et al., 2018). An ex-vivo investigation showed a positive correlation between Treg and butyrate. It indicated that gut microbiota in the NAFLD-HCC model expands the population of total and effector IL-10^+^ Tregs while decreasing the expansion of CD8^+^ cells (Behary et al., 2021). Similarly, it has been verified that IL-2 plays important role in the activation and expansion of CD8^+^ T cells (Chinen et al., 2016). The presence of peripheral Tregs consumes the IL-2, thereby attenuating the functions of CD8^+^ T cells (Chinen et al., 2016). Previous ex-vivo investigation implied that this T cell expression profile is triggered by the gut-microbiota isolated from NAFLD-HCC patients, but not cirrhosis, and demonstrated the microbiota and metabolites-specific regulatory effects on T cells in NAFLD-HCC (Behary et al., 2021). Together, all these investigations highlight the role of Treg in the onset of NAFLD-HCC. However, detailed mechanistic studies are still required to thoroughly understand the functions of Tregs in the pre-tumor process from NAFLD to HCC.

## Conclusion

Chronic hepatic diseases, such as ALD and NAFLD, are emerging as the foremost cause of liver cancer and morbidity worldwide. It has been estimated that the prevalence of NAFLD-associated HCC will drastically increase (up to 45%–130%) in a decade (Grgurevic et al., 2021). The progression of hepatic diseases largely depends on several factors, such as ROS, oxidative stress, lipotoxicity, IR and gut microbiota. In the hepatic microenvironment, numerous hepatic parenchymal, non-parenchymal, innate immune cells, adaptive immune cells, and inflammatory cytokines and chemokines interact with each other to maintain immune homeostasis. However, factors contributing to metabolic steatosis disrupt this homeostasis which greatly affects the population of T cells in the hepatic microenvironment and leads to the CD4^+^ T cell infiltration in the liver. Numerous CD4^+^ T cell subsets participate in regulating the ALD, NAFLD, and NAFLD-related HCC disease progression. Among these T cells, FOXP3^+^ Tregs play pivotal roles in the progression of steatohepatitis and in creating pre-tumor microenvironment settings. It was suggested that during the hepatic injury, factors contributing to the disease progression reduce the activation and development of FOXP3^+^ Tregs. However, an increase in Tregs is involved in tempering the features of ALD and NAFLD by reducing the steatohepatitis and fibrosis, and exerting the immunosuppressive effects by hindering the inflammatory cellular immunity (Albano, 2012; Ikeno et al., 2020). Meanwhile, an increased population of Tregs promotes tumor development by setting a premalignant stage for the progression of HCC in the NASH-associated liver (Wang et al., 2021). It is noteworthy that various parenchymal and non-parenchymal cells in the liver interact to induce an immune response. Increasing evidence demonstrates that various factors influence the activity and function of Tregs. However, studies reporting the impacts of Tregs on the hepatic parenchymal and non-parenchymal cells are not illustrated.

Tregs-targeted therapy is regarded as a prospective HCC therapeutic strategy. So far, numerous innovative therapeutic strategies have been reported to target Tregs in clinical trials. These strategies, including using small molecules or antibodies, disrupt the function or differentiation of Tregs (Tsung and Norton, 2015). Despite advancements in biological and cancer research, little attention has been paid to developing strategies targeting the Tregs, especially in NAFLD-associated HCC. Various effective therapies, including monoclonal antibodies (anti-PD-1, anti-PD-L1, and anti-CTLA-4), have shown favorable prognosis and improved overall survival rates in patients with solid tumous (Saleh and Elkord, 2020a). However, studies demonstrating the effect of these monoclonal antibody usages in treating NAFLD-associated HCC are limited and need attention. More importantly, considering the side effects and low efficacy of these monoclonal antibodies, it is important to identify new anti-tumor drugs and validate the efficacy of newly identified drugs alone or in combination with other immune checkpoint inhibitors. Treg-targeted therapy represses tumor growth by enhancing the infiltration of CD8^+^ cells at the site of the tumor, improving the functions of APCs and minimizing the infiltration of myeloid suppressive cells in TME (Saleh and Elkord, 2019; Saleh and Elkord, 2020b). Thus, realizing the significant effects of the adoptive transfer of Tregs in the NAFLD and NAFLD-related HCC (Van Herck et al., 2020), it is important to evaluate the efficacy of natural or engineered Tregs along with other immune checkpoint inhibitors in NAFLD, premalignant NAFLD and NAFLD-related HCC. Meanwhile, it is worthwhile to understand the effects and function of Tregs in HCC tumors induced by different causative agents and varying disease stages and histological grades.

In conclusion, the cell-cell interactions, production of inflammatory cytokines and chemokines, and antigens in the tumor microenvironment diversify the functional studies of Tregs in HCC tumors. A comprehensive and clear mechanistic understanding of the biology of hepatic Treg cells in the steatosis and premalignant process may lead to inventing novel therapeutic approaches that target Tregs to restrict and treat chronic hepatic diseases, metabolic steatosis and NAFLD-associated HCC.
